# Screening of Heteroaromatic Scaffolds against Cystathionine Beta-Synthase Enables Identification of Substituted Pyrazolo[3,4-c]Pyridines as Potent and Selective Orthosteric Inhibitors

**DOI:** 10.3390/molecules25163739

**Published:** 2020-08-16

**Authors:** Anna-Maria Fantel, Vassilios Myrianthopoulos, Anastasios Georgoulis, Nikolaos Lougiakis, Iliana Zantza, George Lamprinidis, Fiona Augsburger, Panagiotis Marakos, Constantinos E. Vorgias, Csaba Szabo, Nicole Pouli, Andreas Papapetropoulos, Emmanuel Mikros

**Affiliations:** 1 Department of Pharmacy, National and Kapodistrian University of Athens, 157 74 Panepistimiopolis, Zografou, Greece; anmafantel@pharm.uoa.gr (A.-M.F.); vmyriant@pharm.uoa.gr (V.M.); nlougiak@pharm.uoa.gr (N.L.); izantza@pharm.uoa.gr (I.Z.); lamprinidis@pharm.uoa.gr (G.L.); marakos@pharm.uoa.gr (P.M.); pouli@pharm.uoa.gr (N.P.); apapapet@pharm.uoa.gr (A.P.); 2 Department of Biology, National and Kapodistrian University of Athens, 157 01 Panepistimiopolis, Zografou, Greece; tgeorgoulis@med.uoa.gr (A.G.); cvorgias@biol.uoa.gr (C.E.V.); 3 Pharmacology, Section of Medicine, University of Fribourg, Ch. du Musée 18, 1700 Fribourg, Switzerland; fiona.augsburger@unifr.ch (F.A.); csaba.szabo@unifr.ch (C.S.); 4 PharmaInformatics Unit, “Athena” Research and Innovation Center, Artemidos 6, 151 25 Maroussi, Greece

**Keywords:** cystathionine *β*-synthase, hydrogen sulfide, docking-scoring calculations, 7-azido-4-methylcoumarin assay, pyrazolo[3,4-c]pyridine, Sitemap algorithm, back-propagation DNN, thermal shift assay, Bateman module

## Abstract

Cystathionine *β*-synthase (CBS) is a key enzyme in the production of the signaling molecule hydrogen sulfide, deregulation of which is known to contribute to a range of serious pathological states. Involvement of hydrogen sulfide in pathways of paramount importance for cellular homeostasis renders CBS a promising drug target. An in-house focused library of heteroaromatic compounds was screened for CBS modulators by the methylene blue assay and a pyrazolopyridine derivative with a promising CBS inhibitory potential was discovered. The compound activity was readily comparable to the most potent CBS inhibitor currently known, aminoacetic acid, while a promising specificity over the related cystathionine *γ*-lyase was identified. To rule out any possibility that the inhibitor may bind the enzyme regulatory domain due to its high structural similarity with cofactor s-adenosylmethionine, differential scanning fluorimetry was employed. A sub-scaffold search guided follow-up screening of related compounds, providing preliminary structure-activity relationships with respect to requisites for efficient CBS inhibition by this group of heterocycles. Subsequently, a hypothesis regarding the exact binding mode of the inhibitor was devised on the basis of the available structure-activity relationships (SAR) and a deep neural networks analysis and further supported by induced-fit docking calculations.

## 1. Introduction

Endogenously generated hydrogen sulfide, H_2_S, is a signaling molecule of pivotal importance and its biosynthesis in mammalian cells is facilitated by three enzymes, cystathionine *β*-synthase (CBS), cystathionine *γ*-lyase (CSE), and 3-mercaptopyruvate sulfurtransferase (3-MST) [1,2,3,4]. Cystathionine *β*-synthase, or CBS, is the first enzyme of the transsulfuration pathway in which the potentially toxic metabolite homocysteine is converted to cysteine [4]. Among others, human CBS catalyzes the condensation of serine and homocysteine to cystathionine and also utilizes cysteine as a substrate to yield H_2_S. H_2_S is considered as an important biological mediator in a similar fashion to nitric oxide (NO) and carbon monoxide (CO) and CBS is responsible for 20%–75% of total H_2_S production and, more specifically, up to 95% of its production in the brain [5]. The enzyme is located in the central nervous system as well as in other tissues (cardiovascular and gastrointestinal). CBS is regarded as a highly attractive drug target, as the development of potent and selective inhibitors may offer important therapeutic traits for a series of pathological states where H_2_S signaling has a key role [6,7,8,9,10,11]. CBS overexpression has been reported in patients with Down syndrome, and leads to perturbation of H_2_S levels in the human body [12,13]. Interestingly, the CBS gene is located on chromosome 21, the (complete or partial) trisomy which is the genetic basis of the Down phenotype [14,15]. Inhibition of enzymatic activity in these cases can prevent chronic exposure of Down syndrome patients to H_2_S and potential prevention of related neurological deficits [16,17]. Furthermore, CBS up-regulation has been detected in cancer cells including colon cancer, ovarian cancer and lung cancer, amongst others [2,8,18,19,20,21]. Under these conditions, tumor growth is facilitated through stimulation of cellular bioenergetics, cell proliferation and angiogenesis [2,22,23].

In terms of structure, CBS is a unique heme-containing pyridoxal 5′-phosphate (PLP)-dependent enzyme that is allosterically activated by s-adenosylmethionine (AdoMet or SAM) [24,25]. The full-length human CBS is a homotetramer consisting by 63 kDa subunits, with each subunit comprising 551 residues [26]. CBS adopts a three-domain structure encompassing an n-terminal heme-binding domain, a central catalytic domain (PLP cavity) which is accessible only via a narrow channel, and a smaller c-terminal allosteric regulatory domain (CBS motif, Bateman module) [27,28]. The cofactor PLP is deeply buried in a cleft between the n- and the c-terminal domains adjacent to the active site. On the other hand, the heme binding site is located 20 Å away from the active site. Although heme does not contribute to catalysis, it is believed to contribute to redox regulation of CBS. The enzyme regulatory domain is responsible for interaction with SAM. When SAM binds to this domain, it causes allosteric activation of CBS [21,29,30,31]. In mammals, the catalytic activity of CBS is increased up to 5-fold by allosteric SAM binding. In its functional form, CBS is believed to form a domain-swapped dimer where the C-terminal regulatory domain of one subunit is atop the n-terminal catalytic domain of the other (protein data bank code: 1JBQ) [24,32]. The domains of each subunit are reoriented upon SAM binding, leading to the aforementioned five-fold increase in catalytic activity.

A series of different scaffolds showing a promising inhibitory potential against CBS have been reported so far, with many of them being structurally unrelated synthetic molecules or natural products including flavonoids, coumarins and marine metabolites [2,33,34,35,36]. Yet, the most potent CBS inhibitor is a remarkably simple molecule, aminoacetic acid (AOAA), believed to react with PLP toward the formation of a new external aldimine [3,28,37]. However, AOAA is not selective for CBS, as it also inhibits CSE and interacts with other PLP-dependent enzymes as well [9]. Such a simple organic molecule could marginally be considered as a tractable lead for drug development. In this direction, exploring the available chemical space with the objective to discover new scaffolds that can act as promising leads for achieving potent and selective CBS is deemed a highly interesting screening endeavor [38]. The present study describes the evaluation of a unique small molecule library particularly rich in heterocyclic drug-like compounds, the discovery of a pyrazolopyridine derivative as a new selective CBS inhibitor and the elucidation of its inhibitory mode of action. The main results are derived from a series of orthogonal biochemical and biophysical assays and additional support is offered by theoretical approaches, including artificial intelligence modeling and molecular simulations.

## 2. Results and Discussion

### 2.1. Rationale and Compound Selection

**Figure 1:**
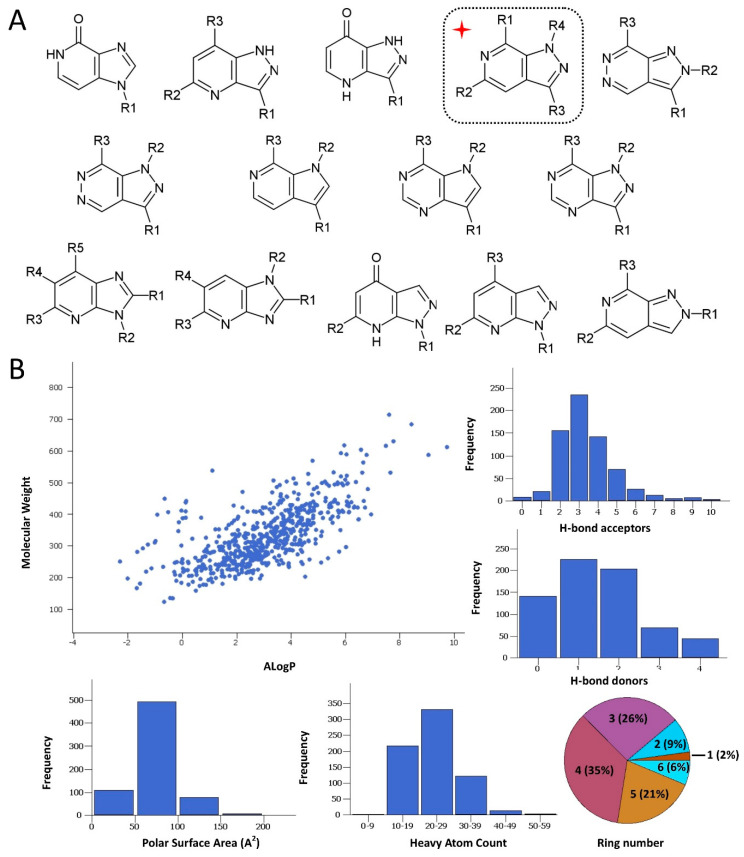
(**A**) The various substituted heteroaromatic scaffolds comprising the screened collection and the pyrazolo[3,4-c]pyridine core identified as a potent cystathionine *β*-synthase (CBS) inhibitor (inset denoted by a red cross). (**B**) Graphical evaluation of the collection drug-likeness in terms of key parameters related with the Rule-of-Five characteristics.

While there is no determined cocrystal structure of CBS with any of the published inhibitors, the available crystal structures and previous screening attempts regarding CBS have offered adequate structural data to support the notion that the protein could be characterized as a druggable target. This is true regardless of the relatively low hit rates and weak-to-medium inhibitory activities that have been so far recorded by the aforementioned screening endeavors against CBS. Though functional CBS comprises a complicated quaternary structure bound to several co-factors by means of their corresponding binding sites, the capacity of efficient ligand binding is deemed as particularly promising as to the active site of the enzyme, which comprises a topologically sound cavity for competitive inhibition by drug-like small molecule binding [10]. This was verified by a druggability analysis of the enzyme active site by implementing the Sitemap algorithm (Schrödinger LLC, New York, NY, USA, 2020). The algorithm characterizes binding sites in terms of structural and geometrical features affecting the affinity of potential small molecule binders. Such features are solvent exposure, degree of protein enclosure, hydrophobicity and hydrophilicity, as well as hydrogen bond capacity. Sitemap derives druggability scores (SiteScore and the variant Dscore), where a SiteScore above 0.8 indicates a druggable site and a value equal or higher than 1 denotes a particularly promising pocket. The analysis afforded a high score (SiteScore: 1.012; Dscore: 0.973), suggesting a predominantly promising cavity and further strengthening the hypothesis that, apart from molecules the size of the natural substrates, the enzyme might also interact with larger molecules that could hence function as modulators. In this direction, a focused compound collection was devised and a screening campaign was undertaken, aiming at the discovery of orthosteric CBS inhibitors. The collection was assembled from the in-house repository of the Pharmacy department of National and Kapodistrian University of Athens (NKUA). The NKUA repository (approximately 2000 entries) is a proprietary compound library assembled and enriched over the last years by synthetic molecules, natural products and semi-synthetic analogues derived from a number of phytochemical and synthetic projects. The library is characterized by high structural originality, as most of its components are natural products isolated from biodiversity hot-spots and their related semisynthetic analogues. In the present study, the objective was to sample the variety of heterocyclic scaffolds that are present in the abovementioned repository at the most efficient and timely fashion. The selection was mainly focused in choosing a representative ensemble of nitrogen-containing heteroaromatic scaffolds that could mimic the natural rings of purine or pyrimidine. Such systems comprise the main core of the repository and, most importantly, fall under regions of chemical space that are widely accepted as highly promising in terms of biological activity and privileged structural character. A number of diverse synthetic heterocycles that could be viewed as purine isosteres were considered, including derivatives that possess a central pyrazolopyridine, pyrrolopyridine, pyrazolopyrimidine, pyrrolopyrimidine, pyrazolopyrazole, pyrazinopyridine and pyrazolopyridazine scaffold (Figure 1A). The collection comprised ~600 molecules of high originality. To achieve higher diversity and increase the success rate, this in-house collection was further enhanced by the addition of top-ranked compounds originating from the National Cancer Institute (NCI) Repository and selected by means of virtual screening. To this end, in silico screening based on rigid docking of the NCI Repository was performed as a part of the current CBS inhibitor development effort. The virtual screen utilized our previously developed model [10] (1JBQ crystal structure of CBS) and Glide (Glide SP algorithm, Schrödinger LLC, New York, NY, USA, 2020) [39,40,41]. In this instance, the absence of any inhibitor-CBS co-crystal structure mandates for manual creation of the docking grid (please see Materials section). The 80 compounds which ranked higher in terms of the GScore scoring function were selected from the NCI Repository and ordered. The final collection (NKUA repository and NCI subset) was characterized by a highly satisfactory degree of drug likeness (Figure 1B), thus enabling efficient exploration of the bioactivity landscape of N-containing heterocycles (analyzed by Canvas, Schrödinger LLC, New York, NY, USA, 2020) [42,43]. More specifically, the majority of the compounds were found to be in overall good compliance with the Rule-of-Five structural features (Figure 1B). As to the structural diversity of the collection, a considerable variety in the side chain decorations and substitution motifs was evident in the number of rings, with 88% of compounds carrying at least one additional ring system apart from the two-ring heteroaromatic core of purine and 1-ring of pyrimidine, respectively.

### 2.2. Protein Expression and In Vitro Evaluations

Expression and purification of CBS have been described as complicated because of the strong tendency of the enzyme to aggregate [44,45,46,47]. In the present study, the protein was expressed and purified as described earlier, whereas specific modifications of the isolation procedures were undertaken as a means to improve both yield and purity of the enzyme. An expression system of the full-length human CBS was constructed and human CBS was expressed as a glutathione S-transferase (GST)-fusion protein [48]. A three-step purification workflow was organized, first by using affinity chromatography (GSTrap FF column), then by changing buffer and pooling high concentration samples into an anion exchange column (Q Sepharose) and, finally, by gel filtration. Consequently, almost 7.5 mg of highly pure full-length active human CBS were obtained from 1 L of *E. coli* overnight culture. As a means to rule out any possibility that the identified hits could interact with the regulatory domain of CBS due to their structural resemblance with SAM (see discussion Section 2.3), the aforementioned domain was also expressed and purified accordingly. The total collection of the in-house heterocyclic structures (~600 molecules) merged with the top-ranked NCI compounds (80 molecules) were evaluated in vitro for their CBS inhibitory potency.

**Figure 2:**
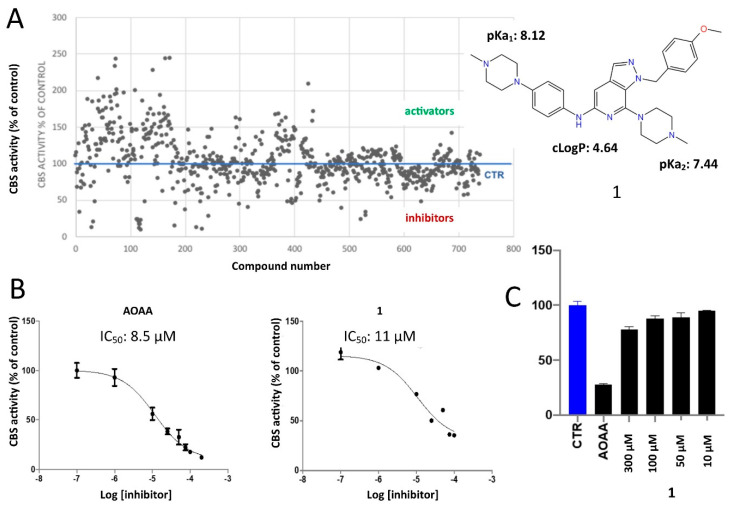
(**A**) A scatter plot summarizing obtained results from the single concentration screen against CBS. (**B**) The dose-response curves of **1** and AOAA show that the inhibitory activity of the pyrazolo[3,4-c]pyridine analogue is comparable to the most potent known CBS inhibitor, aminooxyacetic acid. (**C**) Evaluation of inhibitory potential of **1** against the related enzyme involved in H_2_S production cystathionine γ-lyase (CSE), showing specificity of **1** toward CBS as compared to AOAA.

Several experimental settings, either biochemical or biophysical, have been described for assessing the inhibitory potential of small molecules against CBS. Among them, the methylene blue assay is considered as one of the most robust methods available [49,50,51]. In the present study, enzymatic activity of the CBS fusion protein was measured by the ability to produce H_2_S in a reaction employing l-cysteine and homocysteine substrates. Quantification of H_2_S was performed by using a standard curve and a H_2_S donor. To confirm the adequate complexation of PLP within the protein during the purification process, the assay was performed in the presence and absence of 0.01 mM PLP. In this study, to identify inhibitors, a quick first screening step was performed at a single inhibitor concentration of 50 μM and compounds that afforded higher than 30% CBS inhibition were further validated by additional assays and structurally analyzed. As expected, a moderate hit rate was determined by the screen, whereas several molecules emerged as CBS activators (Figure 2A). However, among the assayed molecules, the pyrazolopyridine derivative **1** was shown to be the most efficacious inhibitor. This specific molecule was previously synthesized in our lab as a potential inhibitor of angiogenesis [52]. In this molecule, the central pyrazolo[3,4-c]pyridine core is substituted by three functional groups which are present in many bioactive analogues, namely a n^1^-4-methoxybenzyl group attached to the pyrazole ring together with a 4-methylpiperazin-1-yl group and a 4-(4-methyl-piperazin-1-yl)phenylamino group connected to the nucleus (Figure 2B). To confirm the biological activity of the newly discovered pyrazolopyridine hit, the IC_50_ value of the inhibitor was determined and directly compared with that of AOAA, calculated in an identical setting. Notably, the dose-response curves were constructed in the presence of 1 mM of l-cysteine and 1 mM homocysteine. The IC_50_ value of **1** was 11 μM, whereas the corresponding value of AOAA was 8.5 μM (Figure 2B). Of interest, when the new inhibitor **1** was tested against the related H_2_S-producing enzyme CSE, it was found to possess considerably lower inhibitory activity (Figure 2C). The pyrazolopyridine inhibitor was tested against GST-CSE in three different concentrations in the presence of 1 mM l-cysteine and 0.01 mM PLP, resulting in no significant inhibitory effect.

For validating the most potent hit identified through the primary screen and rule out any possibility of undesirable interferences with the assay conditions leading to a false positive result, a parallel setting for H_2_S detection by the use of 7-azido-4-methylcoumarin (AzMC) was opted for [53]. The inhibitory effect of **1** on CBS was confirmed with this H_2_S detection method as well, although the IC_50_ value of **1** against CBS by the AzMC assay was determined at 103 μM. This difference likely reflects intrinsic methodological variations between the two assays that turn to be critical when the assayed compounds are ionized with pKa values in very close range to the pH of each setting (pH values: 8.2 for methylene blue; 8 for AzMC assay).

### 2.3. Differential Scanning Fluorimetry

**Figure 3:**
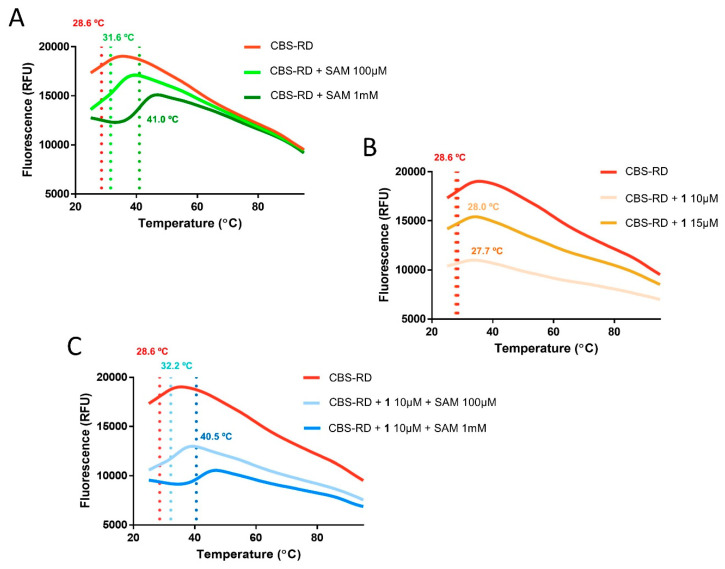
Differential scanning fluorimetry was implemented to identify a possible binding interaction of the inhibitor 1 with the regulatory domain of CBS (CBS-RD). A series of representative melting curves as those shown above, suggest that **1** does not bind CBS-RD. (**A**) Addition of the cofactor s-adenosylmethionine (SAM) (100 μM, light green, *T_m_*: 31.6 °C; 1 mM, dark green, *T_m_*: 41.0 °C) results in a dose-response stabilization effect on CBS-RD as compared to the apoprotein (red, *T_m_*: 28.6 °C). (**B**) In contrast, no stabilization is monitored by increasing concentrations of **1** (10 μM, light brown, *T_m_*: 28.0 °C; 15 μM, dark brown, *T_m_*: 27.7 °C). (**C**) Co-administration of SAM and **1** (100 μM SAM, 10 μM **1**, light blue, *T_m_*: 32.2 °C; 1 mM SAM, 10 μM **1**, dark blue, *T_m_*: 40.5 °C) results in a stabilization and response that are practically identical to the case where only SAM is bound to CBS-RD, showing that no binding or any kind of cooperativity takes place between **1** and the SAM-protein complex.

Even though the whole screening strategy aimed at discovering ligands that target the active site of CBS, the non-negligible resemblance of **1** with the regulatory domain co-factor SAM in terms of their heterocyclic scaffolds prompted the exploration of the possibility that the identified hit inhibits CBS via allosteric binding. To address this issue, differential scanning fluorimetry experiments were undertaken as a way to rule out the possibility for binding interactions between **1** and the regulatory domain of CBS (CBS-RD) [54]. The thermal melt results unambiguously reproduced the previously reported extensive stabilization that cofactor binding offers to the protein, with Δ*T_m_* values showing a dose-response increase in biologically relevant concentrations of SAM (+2.98 °C, 100μM SAM; +12.43 °C, 1 mM SAM; Figure 3A), but failed to show a statistically significant shift for two different concentrations of **1** (10 μM and 15 μM; Figure 3B), whereas addition of both SAM and **1** resulted in melting sigmoidals and respective Δ*T_m_* values that were highly similar to the corresponding of the SAM/CBS-RD system (+3.62 °C, 100 μM SAM, 10 μM **1**; +11.94 °C, 1 mM SAM, 15 μM **1**; Figure 3C) [30]. The lack of stabilization upon thermal denaturation of either CBS-RD or the SAM/CBS-RD complex in the presence of **1** was a clear indication that no significant binding occurs between the inhibitor and the regulatory component of the enzyme, thus providing validity to the suggestion that **1** is an orthosteric CBS inhibitor.

### 2.4. Structure-Activity Relationships, Neural Networks Modeling and Theoretical Simulations

As a means to explore the structure-activity relationships around the newly discovered CBS inhibitory core more thoroughly, a sub-scaffold search was performed and seven derivatives showing high similarity to **1** were recovered from the in-house repository and successively screened (Table 1). Derivatives **2**, **3**, **4** and **8** showed moderate to weak enzyme inhibitory activity when assayed at 100 μΜ and a dose-dependent biological response, whereas analogues **5**, **6** and **7** possessing an aromatic amino substituent at R1 were not active even at high concentrations (Table 1). Although none of those analogues showed an improved activity compared to **1**, the results provided preliminary yet interesting structure-activity relationships that were subsequently used in combination with theoretical simulations to suggest a hypothesis as to the structural basis of CBS inhibition by the aforementioned pyrazolopyridine inhibitors. Minor structural modifications were found to be of major importance for the observed biological activity, as in the case of analogue **2**. This is the second most active inhibitor, showing though a considerable decrease of inhibitory potency from 70% to only 30% at 100 μM that follows a simple removal of the 4-methyl group of the piperazine substituent R1 as compared to **1**. The decrease in activity of **2** was thought of as an indication that the ionization and total charge of studied compounds are factors of key importance for CBS inhibition. More specifically, the structural perturbation involving the transition from **1** to **2** is expected to confer a critical shift not only to the ionization potential of the two compounds, with the basic character of the less potent inhibitor being considerably increased, but also to the trend of protonations given that the emergence of a secondary amine at R1 of **2** reverses the relative order by which the two 4-piperazinyl nitrogens of positions R1 and R2, respectively, are predicted to be ionized (MarvinSketch, ChemAxon). Indeed, in **1** the p*K*_a_ values of the two 4-piperazinyl nitrogens are 7.44 (R1 substituent) and 8.12 (R2 substituent), whereas in **2** the corresponding numbers are 8.84 (R1 substituent, now a secondary amine) and 7.91 (R2 substituent, a tertiary amine). It is also worth noting that the previously described biological activity of a series of pyrazolo[3,4-c]pyridines including **1** and **2** as promising angiogenesis inhibitors was not correlated in terms of structure-activity relationships (SAR) with the CBS-inhibitory potential presented herein [52]. Indeed, the most potent CBS inhibitor **1** was shown to inhibit angiogenesis weakly, whereas **2** characterized by the presence of a 3-phenyl group was a very potent inhibitor of angiogenesis but a marginal inhibitor of CBS.

**Figure 4:**
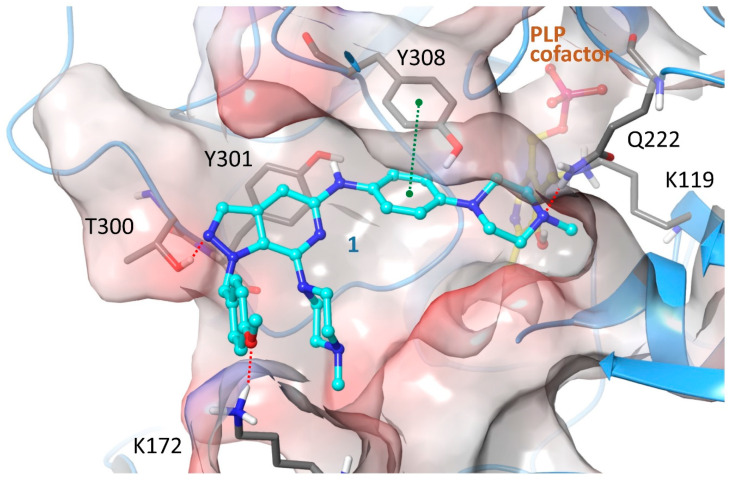
The dominant binding geometry of **1** inside the CBS active site as determined by the induced-fit algorithm for docking calculations. The inhibitor is anchored to the enzyme by a number of hydrogen bonds depicted as red dashed lines, whereas a strong stacking interaction between the phenylamino ring of **1** and Tyr308 (shown as green dashed line) further stabilizes the complex. The two piperazine rings of the inhibitor are deprotonated, whereas piperazine of R2 is positioned in close proximity to the catalytic Lys119, covalently attached in this structure to the cofactor pyridoxal aldehyde as a Schiff base. The protein is depicted as a blue ribbon and molecular surface colored according to the electrostatic potential (red: negative; blue: positive). The ligand and protein interaction geometry demonstrates high complementarity and an excellent occupancy of the pocket by the inhibitor.

In CBS the SAR observations and DSF results seem to favor a hypothesis where the active compounds bind CBS orthosterically and ligand ionization does not contribute to CBS binding through any direct interaction but, conversely, it opposes complex stabilization. Ionization can seriously impact activity either positively, by enhancing binding through charge-assisted hydrogen bonds and electrostatic contacts, or negatively by hindering affinity through repulsions or, more frequently, by the large desolvation penalties involved in ionic ligand binding. With the objective to further explore this scenario, a QSAR model was created by utilizing deep neural networks (DNN) and a dataset comprising the hereby reported and previously published CBS inhibitors (60 actives in total) and 613 inactives. The DNN was constructed using one input layer (243 × 673 points), three hidden layers with 20 neurons each and an output layer with two neurons, namely [0 1] for actives compounds and [1 0] for non-active compounds. The learning workflow was stopped when at least 90% compounds from each group was correctly predicted. The final model resulted in 100% success for active and 94% success for non-active compounds, values that render the prediction significant. The weighting factors between the input and the first hidden layers depict the significance of each descriptor to final decision. Extraction of descriptors with weighting factors greater than 0.8 demonstrated that the most important for group discrimination were those connected to lipophilicity, suggesting it as a key factor for efficient CBS inhibition. This proceeding was in excellent accordance with the hypothesis that the active pyrazolo[3,4-c]pyridine derivatives show a preference to bind CBS as non-ionized molecules. To provide additional strength to the hypothesis, molecular simulations of the interaction between **1** and CBS were undertaken. As previously mentioned, no crystal structure of human CBS has been described in complex with any inhibitor, either substrate-competitive or allosteric. Thus, at present the exact mode of action of known inhibitors is not unambiguously determined, whereas in several cases kinetic results are complex indicating that a mixed-mode inhibition may finally prevail [33]. In this study, in silico experiments were carried out in the catalytic site of the enzyme for estimating the validity regarding the competitive inhibition hypothesis in direct comparison with the SAR notions derived via in vitro screening and, further, for evaluating the contribution of each of the chemical functionalities present in **1** in CBS binding. For enhancing sampling accuracy, induced-fit flexible protein docking (IFD, Schrödinger LLC, New York, NY, USA, 2020) was applied on top-ranked binding geometries determined by rigid docking (Glide, Schrödinger LLC, New York, NY, USA, 2020) [55,56,57,58]. Overall, docking calculations showed that the dominant binding geometry of **1** would agree, yet not unambiguously, with the scenario that charge-assisted interactions are not necessary for efficient **1** binding to CBS (Figure 4). More specifically, the inhibitor binds CBS active site through a network of hydrogen bonds formed between the N1 pyrazole nitrogen of **1** and the side chain hydroxyl of T400, the 4-methoxybenzyl moiety at R4 of the inhibitor and side chain of K172, the phenylamino nitrogen at R2 and Y301, as well as the N4 of piperazine ring at position R1 and the side chain of Q222, whereas the complex is additionally stabilized by a strong π-π stacking contact between the phenylamino ring at R4 and Y308. A clear SAR finding was that replacement of the aliphatic piperazine of R1 by an aromatic ring has a detrimental effect on activity, with the phenylamino analogues **5**, **6** and **7** being inactive. The predicted binding geometry could account for this trend to a good extent. The aforementioned group is positioned in a solvent-exposed and highly polar area of the binding site where the existence of a hydrogen-bond acceptor could favor interaction with the water environment and stabilize the ligand through hydrogen bonds with adjacent polar residues E304 and S147. Moreover, the intramolecular interactions between R1 and R4 substituents are expected to influence free ligand energetics, with stronger stacking in the case of **5**, **6** and **7** likely resulting to a shift toward conformations not favoring protein binding, while an R3 substitution bulkier that H seems unfavorable for CBS inhibition, as **3** carrying a phenyl group at this position retains only a fraction of the activity compared to the otherwise commonly substituted **1**. The presence of Lys172 in a close proximity to the bound inhibitor suggests that a coulombic repulsion between the ionic side chain of Lys172 and the positively charged state of the ligand could be a likely explanation for the increased IC_50_ obtained for **2**, an analogue predicted to be slightly more basic than **1**. It ought to be emphasized though that a less favorable interaction of the cationic form of **1** with the enzyme cannot be excluded. Thus, docking calculations using the IFD protocol and the protonated state of **1** have been performed as well and the most reasonable pose is depicted in Figure S1. Poses where the ligand does not occupy the inner side of CBS active site by a geometry which would permit contact of the inhibitor and PLP were not considered.

To summarize, a rationally designed screen was devised for facilitating the exploration of biologically important subdomains in the chemical space that are represented by purine-like and pyrimidine-like heterocycles. This effort concluded in the discovery of a new scaffold with interesting CBS inhibitory properties. The active pyrazolopyridine analogue showed notable potency toward CBS, whereas a competitive inhibition mode was supported by biophysical analysis. The aforementioned activity was accompanied by moderate selectivity over the related enzyme CSE that is involved in sulfur metabolism as well and individually pursued as a drug target. While this selectivity seemed to be environmentally sensitive, it may provide a rationale and a structural basis for establishing CBS- or CSE-selective inhibition or concurrent CBS-CSE inhibition. Based on molecular simulations, fine-tuning of the ionization potential of the novel scaffold can possibly sustain the design of such enzyme-selective, optimized pyrazolopyridine-based inhibitors. Moreover, as the weak inhibitory properties of the discovered hit toward angiogenesis are likely not related to CBS inhibition, development of dual-specificity analogues seems to be a particularly promising perspective for this lead. To this end, the predictive aspect of the herein derived DNN model is expected to sustain optimization of the compound not only by guiding CBS activity enhancement, but also by rationalizing single- or dual-target specificity requisites. To this respect and in terms of chemistry, the pyrazolo[3,4-c]pyridine scaffold presented herein offers a versatile lead and a highly tractable starting point for developing optimized, biologically active molecules that are in perfect agreement with the principles of druglikeness.

## 3. Materials and Methods

### 3.1. Expression and Purification of Full Length GST-CBS and His Tag-CBS Regulatory Domain

Cystathionine beta synthase cDNA was cloned into the pGEX-Kg vector to create N-terminal GSH-S-Transferase (GST) fusion protein. For expression, *E. coli* expression cells (BL21 CodonPlus (DE3)) were transformed with the expression vector pGEX-Kg/GST-CBS. A fresh colony was chosen to grow a 5 mL starter culture in LB medium overnight at 37 °C and 180 rpm. All media were supplemented with 100 μg/mL ampicillin. The next day, the starter culture was transferred into 1 L of fresh LB medium containing 75 mg/L d-alanine, until cells reach an OD_600_ of 0.6. For induction, isopropyl-beta-d-thiogalactopyranoside (IPTG) was added to a final concentration of 0, 1 mM. The culture was grown overnight at 30 °C and 180 rpm. The cells were subsequently harvested at 6000× *g* for 15 min. After centrifugation, the cell pellet was resuspended in an ice-cold buffer containing 50 mM Tris pH 8.0, 1 mM EDTA, 25 mM DTT, protease inhibitor, 1 mM PMSF, Triton 0.5%, 5 mM PLP and 500 mM NaCl. Cells were lysed by sonication on ice (sonication power ~35%, 20 s pulse, 45 s pause for 20 min). The cell extract was then centrifuged at 14,000× *g* for 30 min at 4 °C. After centrifugation, the soluble fraction containing GST-CBS was loaded onto a GSTrap FF 5 mL affinity column. The column was consecutively washed with five column volumes of binding buffer. The protein was eluted with five volume of elution buffer 50 mM Tris-HCL, 10 mM reduced GSH, pH 8.0. Fractions containing highly pure protein were pooled and immediately applied on anion exchange Q sepharose column fast flow. The column was washed with 20 mM Phosphate buffer pH 8.0, 1 mM EDTA and the bound proteins were eluted with a linear gradient of NaCl from 0 to 1.0 M in the same buffer at a flow rate of 2 mL/min. The protein was further purified by gel filtration (Superose 6 prep. 20 mM Phosphate buffer pH 8.0, 1 mM EDTA, 500 mM NaCl and 10% glycerol) Finally, the GST-CBS protein samples were dialyzed and concentrated in 10 mM Sodium Phosphate Buffer pH 8.2, DTT 1 mM and glycerol 5%. The purity of recombinant enzyme was checked by SDS-PAGE on 12% polyacrylamide gels. The regulatory CBS domain was expressed using the Addgene plasmid #73238 and purified as previously described [30].

### 3.2. Sample Preparation and Library Administration

All tested samples were collected in powder and dissolved in 100% DMSO. The stock solutions were prepared by the use of assay buffer (50 mM Sodium Phosphate pH 8.2) at a final DMSO concentration of 10%. The structural and physicochemical assessment of the collection along with druggability analysis were performed by the use of Canvas software (Schrödinger LLC, New York, NY, USA, 2020).

### 3.3. H_2_S Detection Using the Methylene Blue Assay

H_2_S detection production was measured using the methylene blue colorimetric assay. To test the inhibitory effect of the compounds against CBS, samples were prepared in 100 µL total volume containing 8 μg of recombinant CBS, 1 mM l-cysteine, 1 mM homocysteine, 0.01 mM PLP and 50 mM sodium phosphate buffer pH 8.2. Initially, after sample preparation all samples were incubated at 37 °C for 1 h. After 60 min, the samples were transferred on ice and 1% zinc acetate was added for trapping H_2_S, followed by the addition of 10% trichloroacetic acid for stopping the enzymatic reaction. Afterwards, freshly prepared solutions of N, n-dimethyl-p-phenylenediamine-sulfate in 7.2 M HCl and FeCl_3_ in 1.2 M HCl were added followed by 15 min in the dark resulting in the formation of blue color. The sample solutions were transferred in transparent 96-well flat blank plates and the absorbance was measured at 650 nm. H_2_S quantification was carried out by a standard curve of Na_2_S (0–250 µM).

### 3.4. H_2_S Detection Using the 7-Azido-4-Methylcoumarin Assay

Using 50 mM Tris HCl pH 8 solution and black 96-well plate, 0.5 µg/well of recombinant CBS was incubated 1 h at 37 °C in presence of various final concentrations of **1** in a total volume of 100 µL. CBS substrates were then added to reach 200 µL total volume, 2 mM l-cysteine, 2 mM homocysteine, 0.005 mM PLP and 0.5 mM SAM, as well as the probe 7-azido-4-methylcoumarin (AzMC) (Sigma-Aldrich, Saint Louis, MO, USA) at a final concentration of 10 µM (pH 8.0). Fluorescence was measured in kinetic mode at 37 °C with an Infinite 200 Pro reader (Tecan), with excitation and emission wavelengths of 365 nm and 450 nm, respectively. The IC_50_ of the inhibitor was calculated using GraphPad Prism nonlinear fitting curve function.

### 3.5. Differential Scanning Fluorimetry

The regulatory domain of CBS was assayed at 2 μM in a buffer consisting of 10 mM HEPES at pH 7.8, 150 mM NaCl and 10× SYPRO orange. Ligand concentrations of 10 μM and 15 μM were assessed, while all experiments were performed without SAM or in the presence of SAM at 100 μM or 1 mM. The BioRad CFX-Connect RT-PCR instrument and white BioRad 96-well plates were utilized. Relative fluorescence intensities were measured by increasing the temperature from 25 °C to 95 °C at 0.5 °C/min and the melting curves along with *T*_m_ values were calculated by non-linear fitting of fluorescence units over temperature by using a four-parameter logistic function as provided in GraphPad Prism v. 7 software.

### 3.6. Deep Neural Networks

The Deep Neural Network (DNN) algorithm employed is back propagation (BP). The dataset was based on 673 compounds tested in vitro and was divided to two subsets. The first contained all molecules (*n* = 60) showing more than 50% inhibition, labeled as active compounds. The rest of molecules (*n* = 613) were labeled non-active compounds. Topological descriptors were calculated from the molecular descriptors workflow as implemented in Schrodinger Suite 2019. (Schrödinger LLC, New York, NY, USA, 2020). Initially 277 topological descriptors were predicted. All descriptors with zero values for all molecules were removed, reducing the final number to 243. The values of each descriptor were normalized using the unit variance procedure. The training parameters for the DNN model were set as following: the number of hidden layers was 3; the number of neurons in each hidden layer was 20; the number of neurons in the output layer was 2, [1 0] for active compounds and [0 1] for non-active compounds; the activation and transfer functions were both sigmoid function; all weights of the network were initialized as random values; the number of iterations ranged from 1000 to 5000; during the gradient descent optimization procedure, the learning rate was 0.01. All mathematical calculations were run using the software package MATLAB v2018b developed by MathWorks, USA (http://www.mathworks.com). The descriptors with greater importance were those connected with lipophilicity, partial equalization of orbital electronegativity, and connectivity indexes such as Balaban-type connectivity index J.

### 3.7. Molecular Simulations

The CBS crystal structure deposited under PDB code 1JBQ was selected and prepared for calculations, utilizing the Protein Preparation module of Maestro software (Schrödinger LLC, New York, NY, USA, 2020). The docking grid was prepared accordingly and centered manually around three reference points in the active site of CBS, namely the cofactor PLP and the two active site residues Tyr223 and Gly307. A stepwise workflow was followed, with rigid docking calculations (Glide SP algorithm, Schrödinger LLC, New York, NY, USA, 2020) deriving ensembles of low-energy binding geometries for the studied compounds and the induced-fit algorithm (IFD, Schrödinger LLC, New York, NY, USA, 2020) implemented for optimally exploring the exact binding site of the most interesting analogues in terms of a flexible protein representation. Marvin was used for drawing, displaying and characterizing chemical structures, substructures and reactions, Marvin v 17.13.0, 2017, ChemAxon (http://www.chemaxon.com). The assessment of ionization constants for the compounds was accomplished by MarvinSketch by using the macro mode and static prefix parameters and considering tautomerization and resonance.

### 3.8. Statistical Analysis

Statistics and graphs were created by the use of GraphPad Prism version 8.0. Evaluation and statistical analysis of in vitro screening experiments are represented as the means ± SD. Furthermore, one-way ANOVA multiple comparison test was performed and *p*-values less than 0.05 were considered statistically significant.

## Figures and Tables

**Table 1:**
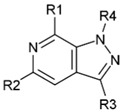
The inhibitory activity of the 8 pyrazolo[3-c], pyridine derivatives against full length CBS. Percentage enzyme inhibition was measured at a final inhibitor concentration of 100 μM.

Compound	R1	R2	R3	R4	Inhibition (%)
**1**	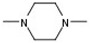	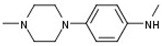	H	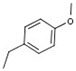	70
**2**	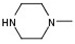	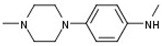	H	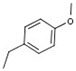	30
**3**	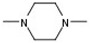	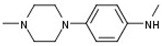	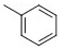	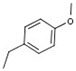	20
**4**	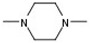	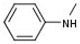	H	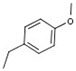	20
**5**	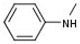	N≡C	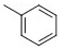	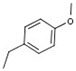	No inhibition
**6**	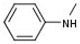	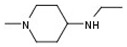	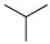	H	No inhibition
**7**	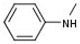	N≡C	H	H	No inhibition
**8**	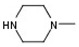	Cl	H	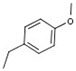	<20

## Supplementary Materials

The Supplementary Materials are available online.
