# Supplementary material for: Screening of Heteroaromatic Scaffolds against Cystathionine Beta-Synthase Enables Identification of Substituted Pyrazolo[3,4-c]Pyridines as Potent and Selective Orthosteric Inhibitors
Source: Molecules. 2020 Aug 16;25(16):3739. doi: 10.3390/molecules25163739 (PMC7465669; doi:10.3390/molecules25163739)
Supplement: Supplementary file 1 [file molecules-25-03739-s001.pdf]

Screening of heteroaromatic scaffolds against Cystathionine beta-synthase enables identification of substituted pyrazolo[3,4-c]pyridines as potent and selective orthosteric inhibitors

Anna-Maria Fantel[1], Vassilios Myrianthopoulos[1], Anastasios Georgoulis[2], Nikolaos Lougiakis[1], Iliana Zantza[1], George Lambrinidis[1], Fiona Augsburger[3] Panagiotis Marakos[1], Costantinos E. Vorgias[2], Csaba Szabo[3], Nicole Pouli[1], Andreas Papapetropoulos[1] and Emmanuel Mikros[1,4*]

[1] Department of Pharmacy, National and Kapodistrian University of Athens, Athens, Greece; anmafantel@pharm.uoa.gr; vmyriant@pharm.uoa.gr; nlougiak@pharm.uoa.gr ; izantza@pharm.uoa.gr; lamprinidis@pharm.uoa.gr; marakos@pharm.uoa.gr; pouli@pharm.uoa.gr; apapapet@pharm.uoa.gr; mikros@pharm.uoa.gr

[2] Department of Biology, National and Kapodistrian University of Athens, Athens, Greece; tgeorgoulis@med.uoa.gr; cvorgias@biol.uoa.gr

[3] Chair of Pharmacology, Section of Medicine, University of Fribourg, Fribourg, Switzerland; fiona.augsburger@unifr.ch; csaba.szabo@unifr.ch

[4] PharmaInformatics Unit, "Athena" Research and Innovation Center; Athens, Greece; mikros@pharm.uoa.gr

* Correspondence: mikros@pharm.uoa.gr; Tel.: +30 2107274813

SUPPLEMENTARY MATERIAL

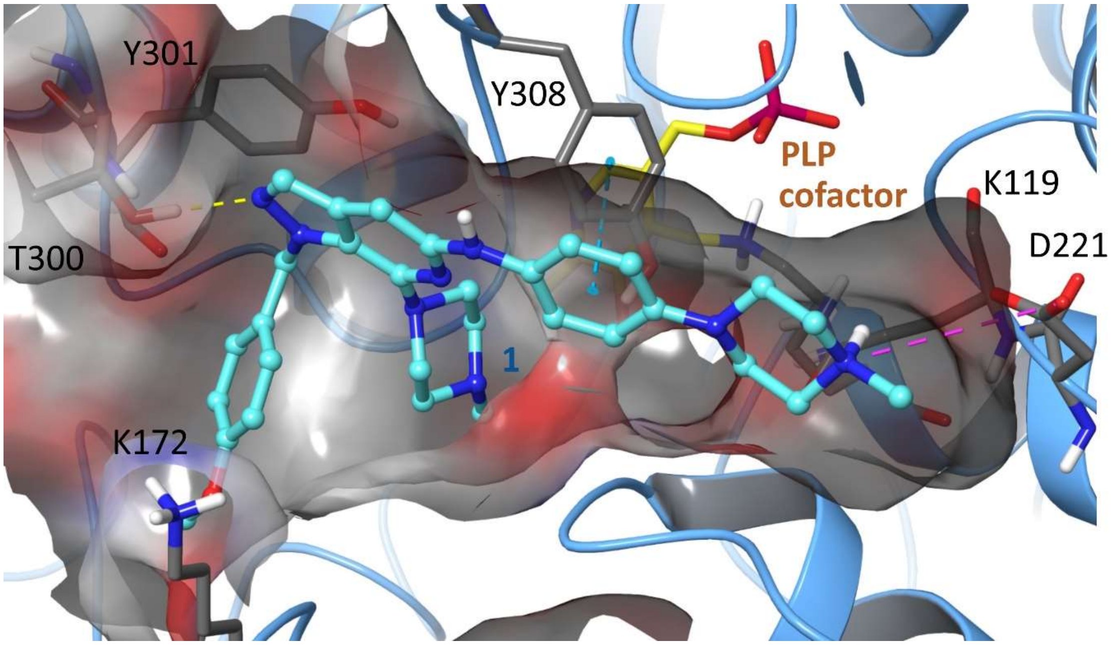

Figure S1. The dominant binding geometry of the protonated **1** inside the CBS active site as determined by the induced-fit algorithm for docking calculations. The inhibitor is mainly anchored to the enzyme by a hydrogen bond depicted as a yellow dashed line between the side chain of Thr300 and the inhibitor pyrazole ring, whereas a salt bridge (magenta dashed line) is formed between the protonated N4 of piperazine and the side chain of Asp221. A stacking interaction between the phenylamino ring of **1** and Tyr308 (shown as blue dashed line) further stabilizes the complex. The protein is depicted as a blue ribbon and molecular surface colored according to the electrostatic potential (red: negative; blue: positive).
